# SEM, EDS and XPS Analysis of the Coatings Obtained on Titanium after Plasma Electrolytic Oxidation in Electrolytes Containing Copper Nitrate

**DOI:** 10.3390/ma9050318

**Published:** 2016-04-27

**Authors:** Krzysztof Rokosz, Tadeusz Hryniewicz, Dalibor Matýsek, Steinar Raaen, Jan Valíček, Łukasz Dudek, Marta Harničárová

**Affiliations:** 1 Division of Surface Electrochemistry & Technology, Faculty of Mechanical Engineering, Koszalin University of Technology, Racławicka 15-17, PL 75-620 Koszalin, Poland; rokosz@tu.koszalin.pl (K.R.); Lukasz.Dudek@tu.koszalin.pl (Ł.D.); 2 Institute of Geological Engineering, Faculty of Mining and Geology, ŠB—Technical University of Ostrava, 708 33 Ostrava, Czech Republic; dalibor.matysek@vsb.cz; 3 Department of Physics, Norwegian University of Science and Technology (NTNU), Realfagbygget E3-124 Høgskoleringen 5, NO 7491 Trondheim, Norway; steinar.raaen@ntnu.no; 4 Institute of Physics, Faculty of Mining and Geology, VŠB—Technical University of Ostrava, 708 33 Ostrava, Czech Republic; jan.valicek@vsb.cz (J.V.); marta.harnicarova@vsb.cz (M.H.); 5 Institute of Clean Technologies for Mining and Utilization of Raw Materials for Energy Use, Faculty of Mining and Geology, VŠB—Technical University of Ostrava, 708 33 Ostrava, Czech Republic; 6 Regional Materials Science and Technology Centre, Faculty of Metallurgy and Materials Engineering, VŠB—Technical University of Ostrava, 708 33 Ostrava, Czech Republic

**Keywords:** titanium, plasma electrolytic oxidation (PEO), micro arc oxidation (MAO), copper nitrate, Scanning Electron Microscopy (SEM), Energy Dispersive X-ray Spectroscopy (EDS), X-ray Photoelectron Spectroscopy (XPS)

## Abstract

In the paper, the Scanning Electron Microscopy (SEM) with Energy Dispersive X-ray Spectroscopy (EDS) and X-ray Photoelectron Spectroscopy (XPS) results of the surface layer formed on pure titanium after plasma electrolytic oxidation (micro arc oxidation) at the voltage of 450 V are shown. As an electrolyte, the mixture of copper nitrate Cu(NO_3_)_2_ (10–600 g/L) in concentrated phosphoric acid H_3_PO_4_ (98 g/mol) was used. The thickness of the obtained porous surface layer equals about 10 μm, and it consists mainly of titanium phosphates and oxygen with embedded copper ions as a bactericidal agent. The maximum percent of copper in the PEO surface layer was equal to 12.2 ± 0.7 wt % (7.6 ± 0.5 at %), which is the best result that the authors obtained. The top surface layer of all obtained plasma electrolytic oxidation (PEO) coatings consisted most likely mainly of Ti_3_(PO_4_)_4_∙nH_3_PO_4_ and Cu_3_(PO_4_)_2_∙nH_3_PO_4_ with a small addition of CuP_2_, CuO and Cu_2_O.

## 1. Introduction

Nowadays, metallic biomaterials, such as titanium [1,2], niobium [3], zirconium [4], tantalum [5], as well as their alloys [6,7,8], are very often used because of their good characteristics, including immunity to corrosion and excellent biocompatibility. In order to improve the biological performance of the material, it is appropriate to use specific surface processing methods. Additional surface electrochemical treatments, such as electropolishing [2,3,9,10,11,12,13,14,15,16] and plasma electrolytic oxidation [1,4,5,6] for the metallic surface layer modification, are used in order to improve the tissue implant connection. In addition, it should be noted that, in order to protect the tissue against infection, the silver [17,18,19,20] or copper [21,22,23] ions are implemented into the surface layer.

Many of the previous studies of PEO have been carried out on titanium under a relatively wide range of conditions for the preparation of the coatings. They are: different electrical regimes, treatment times and electrolyte compositions. The most common electrolytes used for plasma electrolytic oxidation of titanium are: phosphoric and sulfuric acids [24], sodium metasilicate pentahydrate [25], silicon acetate within Ca-β-glycerophosphate and NaOH [26], Ca-β-glycerophosphate and calcium acetate [27], potassium phosphate with potassium hydroxide [28], potassium pyrophosphate and potassium hydroxide [28], calcium glycerophosphate with calcium acetate [29], tripotassium phosphate and potassium hydroxide with and without monoclinic zirconia powder [30], sodium silicate with phosphoric acid and potassium hydroxide [31], tungstosilicic acid [32], ethylene diamine tetraacetic acid disodium with calcium oxide and calcium dihydrogen phosphate and sodium metasilicate nonahydrate [33], sodium phosphate with hydrated sodium borate and sodium tungstate dihydrate and iron(III) oxalate [34], sodium hydroxide with monosodium dihydrogen orthophosphate with and without Cu nanoparticles [35], β-glycerophosphate disodium salt pentahydrate with calcium acetate hydrate [36], calcium acetate hydrate with disodium hydrogen phosphate anhydrous [37]. On the other hand, it must be pointed out that the current used for that treatment may be direct [24,25,26,27,28,29,32,34,35,36] or alternating [30,31], as well as pulsed [33,37,38] current.

This work has been performed to reveal a new method of electrochemical surface treatment allowing for an improved biocompatibility of the treated biomaterial. For the studies, a plasma electrolytic oxidation (PEO) technology was used to treat titanium of a commercial purity. Such an obtained surface layer was investigated by means of Scanning Electron Microscopy (SEM), Energy Dispersive X-ray Spectroscopy (EDS), and X-ray Photoelectron Spectroscopy (XPS) methods.

## 2. Method

### 2.1. Material, Electrolyte and Setup

The plasma electrolytic oxidation of commercial purity titanium (CP Ti Grade 2) was performed at a voltage of 450 V for 3 min. The main elements of the setup were: a processing cell, a three-phase transformer with Graetz bridge, the electrodes and connecting wiring. The scheme of the set-up used was presented earlier in [38]. The titanium samples of dimensions of 30 × 10 × 1 mm were employed for the studies. Current density for constant voltage was not recorded, but it was observed to decrease over time. A cylinder made of AISI 316L stainless steel was used as a cathode.

For the studies, a mixture of 1000 mL of concentrated orthophosphoric H_3_PO_4_ acid (85%) with 10 up to 600 g/L of copper nitrate Cu(NO_3_)_2_ electrolytes, were used. For each run, the electrolytic cell made of glass was used, containing up to 500 dm^3^ of the electrolyte solution.

### 2.2. Set Ups for SEM and EDS

Scanning electron microscope (SEM), FEI Quanta 650 FEG (Field Electron and Iron Company (FEI), Hillsboro, OR, USA), equipped with energy-dispersive X-ray spectroscopy (EDS, FEI), for surface analysis was used. The microscope operated under the following conditions: voltage 15 kV, current 8–10 nA, beam diameter 6 µm, decreased vacuum in the chamber with the pressure of 50 Pa. The identification of spectral lines was performed by means of a spectral decomposition with holographic deconvolution using a targeted peak deconvolution function. All of the EDS data obtained by Statistica 10 [39] were processed.

### 2.3. XPS Studies

The XPS measurements on oxidized titanium samples were performed by means of the SCIENCE SES 2002 instrument (SCIENTA AB, ScientaOmicron, Uppsala, Sweden) using a monochromatic (Gammadata-Scienta) Al Kα (hν = 1486.6 eV) X-ray source (18.7 mA, 13.02 kV). Scan analyses were carried out with the measurement area of 3 × 1 mm^2^ and a pass energy of 500 eV with the energy step of 0.2 eV and the step time of 200 ms. The binding energy of the spectrometer has been calibrated by the position of the Fermi level on a clean metallic sample. The power supplies were stable and of high accuracy. The experiments were carried out in an ultra-high-vacuum system with a base pressure of about 6 × 10^−10^ Pa. The XPS spectra were recorded in normal emission. For the XPS analyses, the Casa XPS 2.3.14 software (Shirley background type [11]) was used. All binding energy values presented in this paper were charge corrected to C 1s at 284.8 eV. All XPS spectra of titanium (Ti 2p), phosphorus (P 2p), copper (Cu 2p), as well as oxygen (O 1s) and carbon (C 1s) with 9 sweeps were performed. Additionally, for the copper Cu 2p spectra, the measurements were repeated by 36 sweeps to increase the signal-to-noise ratio to 6. For the interpretation of the deconvoluted spectra, the literature positions [40,41] were applied.

## 3. Results

**Figure 1:**
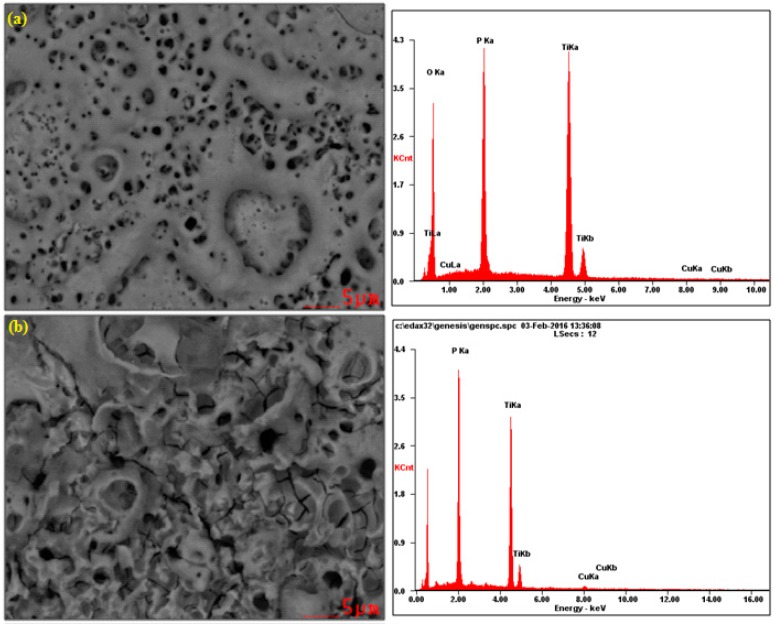

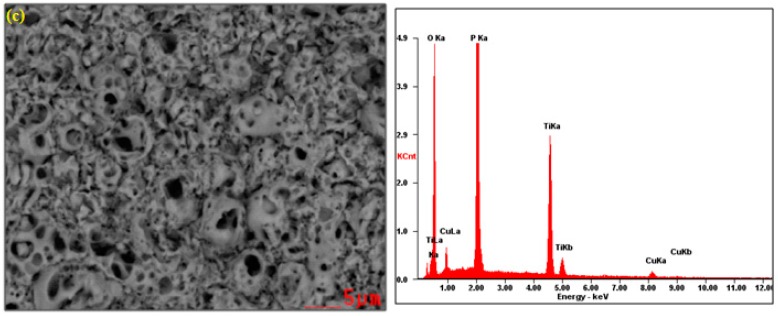
SEM and EDS results of titanium surface after PEO at a voltage of 450 V in the electrolyte consisting of 1 L H_3_PO_4_ with: (**a**) 10 g/L; (**b**) 300 g/L; (**c**) 600 g/L Cu(NO_3_)_2_.

In Figure 1, the SEM and EDS results of the surface layer formed on titanium after PEO at the voltage of 450 V in electrolyte consisting of 1 L H_3_PO_4_ with the addition of (1) 10 g/L; (2) 300 g/L and (3) 600 g/L of Cu(NO_3_)_2_ are presented. All SEM photos show that the obtained surfaces are porous. Additionally, on the surface treated in the electrolyte consisting of 300 g/L of copper nitrate, the cracks in the surface layer are visible beyond the pores, which may be indicative of too rapid growth in volume of the layer. Interestingly, the increasing of the copper nitrate up to 600 g/L does not result in such cracks. Most likely, the copper ions inserted in the porous phosphorus-oxygen-titanium structure create the original modified surface layer. On the basis of EDS peaks, it can be concluded that, with the increase of copper nitrate in concentrated phosphoric acid, the amount of titanium in the surface layer decreases, whereas the increase of phosphorus and copper amounts is observed.

**Figure 2:**
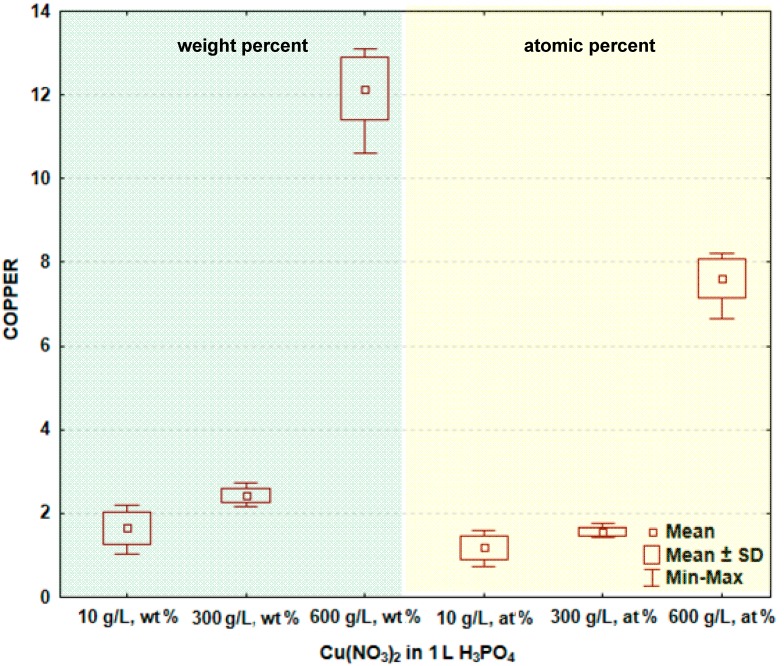
Box and whisker plots of the amount of copper in the surface layer formed on titanium after PEO. SD: Standard deviation.

In Figure 2 and Table 1, the box and whisker plots with the descriptive statistics of the amount of copper in the surface layer formed on titanium after PEO are presented, respectively. One may notice that the copper amount in the surface layer increases with increasing the content of copper nitrate in the electrolyte. The minimum amount of copper in the surface layer was detected after the PEO performed in the electrolyte containing 10 g/L Cu(NO_3_)_2_ and was equal to 1.7 ± 0.4 wt % (1.2 ± 0.3 at %), and the maximum was found in the electrolyte containing 600 g/L Cu(NO_3_)_2_ in H_3_PO_4_, and equaling 12.2 ± 0.7 wt % (7.6 ± 0.5 at %). The significance tests in Statistica software (Version 10) [39] for all data were performed and show that the amounts of copper in the surface layer formed on titanium after PEO in the electrolytes containing various amounts of copper nitrate belong to different data populations. Therefore, it is possible to conclude that there are significant differences in the copper amount in the passive layer between the treated samples.

**Figure 3:**
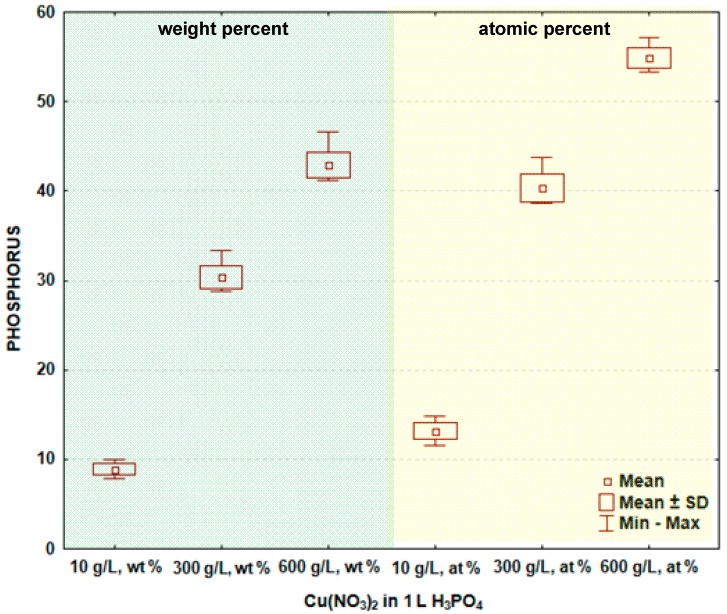
Box and whisker plots of the amount of phosphorus in surface layer formed on titanium after PEO.

In Figure 3 and Table 2, the box and whisker plots with the descriptive statistics of the amount of phosphorus in the surface layer formed on titanium after PEO are shown, respectively. It is visible that the phosphorus amount (similar as copper) increases with the increase of the copper nitrate in the electrolyte. The minimum amount of phosphorus in the surface layer was detected after the PEO performed in the electrolyte containing 10 g/L Cu(NO_3_)_2_ and was equal to 8.9 ± 0.6 wt % (13.2 ± 0.9 at %), and the maximum was found for 600 g Cu(NO_3_)_2_ in 1000 mL H_3_PO_4_, equaling 42.9 ± 1.4 wt % (54.9 ± 1.1 at %). The significance tests presented in Table 2 show that the amounts of phosphorus in the surface layer formed on titanium after PEO in the electrolytes containing various amounts of copper nitrate belong to different data populations. Following that, it is possible to conclude that there are significant differences in the phosphorus amount in the passive layer between the treated samples. The study shows that the increasing concentration of copper nitrate in the phosphoric acid results in the increase of both copper, as well as phosphorus in the surface layer. These phenomena can be explained by the formation of chemical compounds between titanium, phosphorus, copper and oxygen, which are insoluble in the concentrated phosphoric acid.

**Figure 4:**
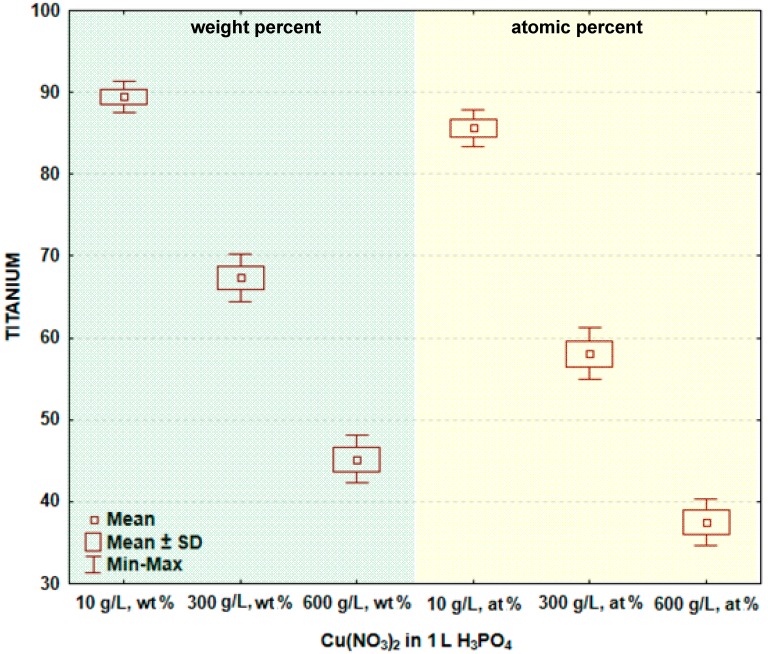
Box and whisker plots of the amount of titanium in the surface layer formed on titanium after PEO.

In Figure 4 and Table 3, the box and whisker plots with the descriptive statistics of the amount of titanium in the surface layer formed on titanium after PEO are given, respectively. The maximum amount of titanium in the surface layer was detected after the PEO done in the electrolyte containing 10 g/L Cu(NO_3_)_2_ and was equal to 89.4 ± 0.9 wt % (85.6 ± 1.1 at %), whereas the minimum was found at 600 g/L Cu(NO_3_)_2_, equaling to 42.2 ± 1.5 wt % (37.5 ± 1.4 at %). This may suggest that for 10 g/L Cu(NO_3_)_2_ in one liter of phosphoric acid, the coating is much thinner than the one formed in the electrolyte within 600 g/L of copper nitrate. The significance tests presented in Table 3 show that the amounts of oxygen in the surface layer formed on titanium after PEO in the electrolytes containing various amounts of copper nitrate mostly do not belong to different data populations. That finding allows for the conclusion that generally, there are no significant differences in the oxygen amounts in the passive layer between the treated samples.

**Figure 5:**
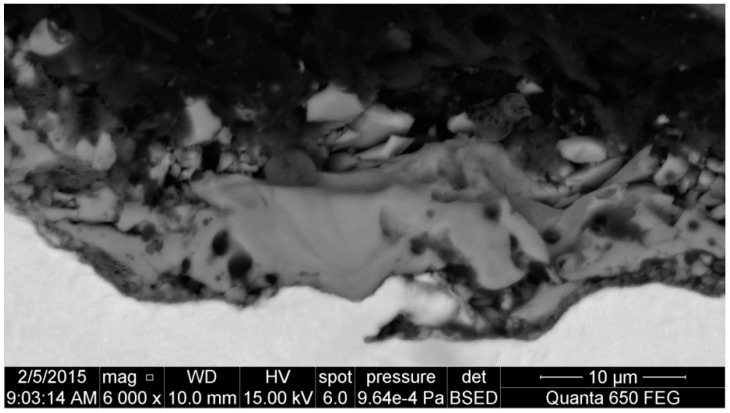
Cross-section of the PEO surface layer formed on titanium after PEO at 450 V.

For example, Figure 5 presents a cross-section of the surface layer obtained on pure titanium after PEO at the potential of 450 V in the electrolyte consisting of 300 g of copper nitrate dissolved in 1000 mL of concentrated phosphoric acid (98 g/mol). One may easily notice that the obtained surface layer is heterogeneous and porous, and its thickness is equal to about 10 μm. Additionally, one may presume that it is more biocompatible than a pure titanium surface and can be used as a transition layer between the titanium biomaterial and tissue.

To find out the chemical composition of coatings formed during the PEO process, the XPS measurements were performed. With the help of that method, the oxidation stages of inter alia titanium, phosphorus and copper were possible to find.

**Figure 6:**
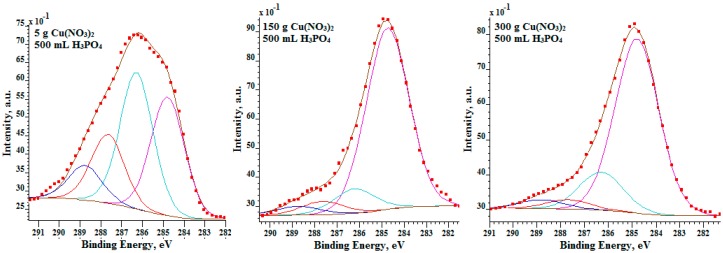
XPS high resolution C 1s spectra of surface layer formed on titanium after PEO.

In Figure 6, the fittings of C 1s spectra are shown. With this analysis, it is possible to determine how much oxygen is bound to the carbon contamination nano-layer and how much of this is bound with other chemical elements of the passive surface layer. All C 1s spectra were deconvoluted by four peaks: C–C/C–H (284.8 eV), C–O–H/C–O–C (286.3 eV), C=O (287.7 eV), O–C=O (288.8 eV). On the basis of the obtained results, it was possible to separate oxygen bonded with carbon (contamination carbon layer) and with the chemical elements contained in the passive layer.

In Table 4, there are enlisted results of the passive PEO layer obtained on pure CP titanium after the electrochemical plasma oxidation (PEO) in the electrolyte consisting of Cu(NO_3_)_2_ and H_3_PO_4_. It was found that in the PEO layer (of about 10-µm thick), mainly the titanium-copper phosphate compounds are visible. Assuming that all of the oxygen in the passive layer is bonded with phosphorus to form phosphates, then one may presume that not all of the phosphorus was used. In all of the PEO layers, a minimum of 7 at % of the phosphorus, which was not bonded with the oxygen, was found. Hence, the phosphorus must form anaerobic compounds with the titanium or copper. Based on Table 4, it can be noticed that the amount of copper in the PEO layer increases very slowly, but regarding the bactericidal properties, its amount is quite satisfactory. It should be noted that the XPS measurements allow one to study only the first 10 nm of the PEO layer; however, in accordance with the recent results revealed in [1], its thickness may be of a few micrometers.

**Figure 7:**
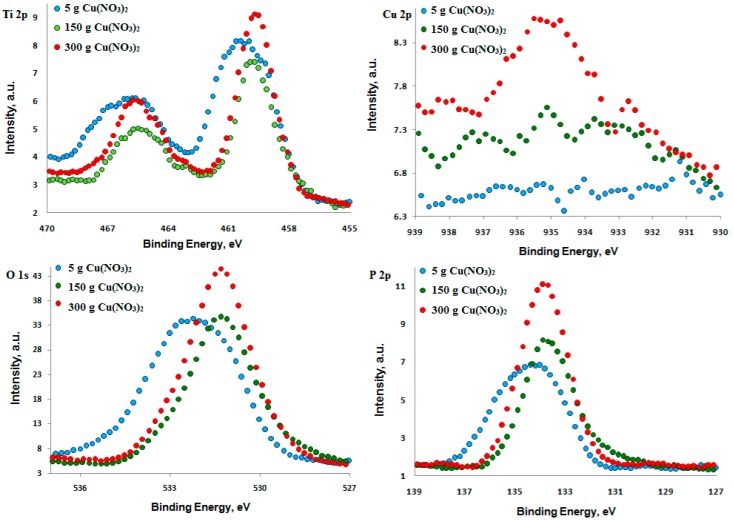
XPS high resolution Ti 2p, Cu 2p, O 1s, and P 2p spectra of surface layer formed on titanium alloy after PEO.

In Figure 7, the XPS high resolution spectra of titanium (Ti 2p), copper (Cu 2p), oxygen (O 1s) and phosphorus (P 2p) are presented. Concerning these results, it is possible to predict, with a high probability, what chemical compounds of titanium were formed after the PEO treatment. The P 2p spectra indicate that in the surface layer formed after the electrochemical PEO treatment with the addition of 300 g/L and 600 g/L Cu(NO_3_)_2_ to 1 L H_3_PO_4_, the highest/main peak has binding energy equal to about 133.9 eV, suggesting the presence of phosphates (PO_4_^3−^) in the studied surface layer. In the case of oxidation in the electrolyte containing 10 g/L Cu(NO_3_)_2_ in 500 mL H_3_PO_4_, apart from PO_4_^3−^, an additional peak is visible, whose binding energy is about 135.2 eV. That can suggest the presence of H_3_PO_4_ acid molecules in the surface layer. In the case of Ti 2p_3/2_ spectra, the maximum binding energies are noted in the points 460.4 eV, 459.9 eV, 459.6 eV referred to 10 g/L, 300 g/L, 600 g/L Cu(NO_3_)_2_ in 1000 mL H_3_PO_4_, respectively. The Ti 2p_3/2_ spectrum combined with P 2p spectrum can suggest that the titanium detected in the surface layer is on the fourth oxidation stage and most likely appears as the Ti_3_(PO_4_)_4_∙nH_3_PO_4_ compound. After the PEO oxidation in the electrolyte containing lower contents of the copper nitrate (10–300 g/L) in the phosphoric acid (1000 mL), the XPS signal of Cu 2p from the surface layer is on the noise level. Only by increasing the amount of the copper nitrate up to 600 g per 1000 mL of phosphoric acid, a distinct spectrum of Cu 2p was recorded.

**Figure 8:**
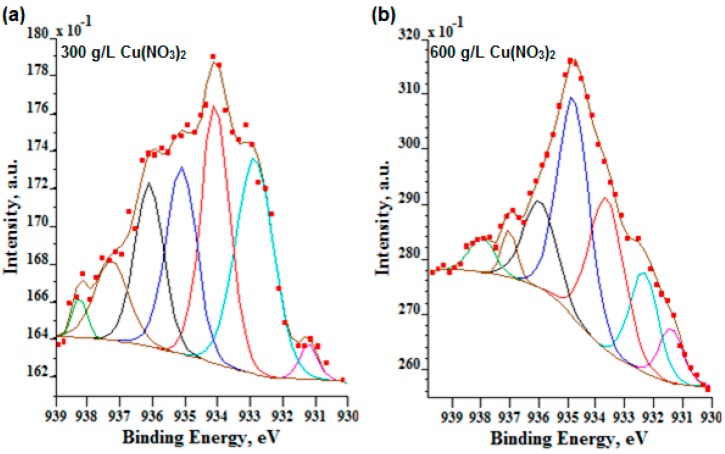
High XPS resolution Cu 2p_3/2_ spectra within their fitting of the surface layer formed on titanium after PEO in the electrolyte containing 300 g/L (**a**) and 600 g/L (**b**) of Cu(NO_3_)_2_ in H_3_PO_4_.

In the case of a low signal-to-noise ratio, *i.e.*, three for nine sweeps (Figure 8), the authors decided to repeat XPS high resolution scans for the Cu 2p_3/2_ region with 36 sweeps (signal-to-noise ratio equals six). Based on the obtained results, which are depicted in Figure 8, it should be noted that for the lowest amount of copper nitrate (10 g/L) in phosphoric acid, the signal is still very noisy; hence, it was not possible to perform the correct fitting.

In Figure 8 and Table 5, the high resolution Cu 2p_3/2_ spectra for two high signals (300 g and 600 g of Cu(NO_3_)_2_ per 1000 mL H_3_PO_4_) are presented. The two first peaks at 932.3 eV and 932.9 eV (Figure 8a) and 932.4 eV (Figure 8b) and for both analyzed electrolytes can be interpreted most likely as CuP_2_ and Cu_2_O, respectively. Other peaks of the ingrown energies are also responsible for the presence of Cu^2+^, *i.e.*, 933.6, 934.1 eV are responsible mostly for CuO; 934.8, 935.1, 936.1, 937.2, 938.1 eV peaks may be responsible for the presence of Cu^2+^, most likely as the CuO∙Cu_3_(PO_4_)_2_∙nH_3_PO_4_ compound in the PEO layer. Following that path, it should be noted that the more copper nitrate in electrolyte, the more anaerobic (CuP_2_) and aerobic phosphorus-copper compounds (Cu_3_(PO_4_)_2_∙nH_3_PO_4_) are in the PEO surface layer.

The authors have also calculated the Cu/P, Cu/Ti and P/Ti ratios, and they are presented in Table 6. The PEO layer has been characterized by the dimensionless numbers. This is important because they can be used for the comparison of other surface layers containing phosphorus and copper incorporated.

## 4. Conclusions

The plasma electrolytic oxidation of titanium as a biomaterial at a voltage of 450 V in electrolyte consisting of copper nitrate and concentrated phosphoric acid allows one to obtain the porous surface layer with the thickness of some micrometers. The most important fact is that inside of the surface layer, there are embedded copper ions, which are known to have a bactericidal effect. The biggest achievement of the work is gaining the percent of copper in the porous PEO surface layer reaching up to 12.2 ± 0.7 wt % (7.6 ± 0.5 at %), which should be considered as a good result. Increasing the copper nitrate amount in the solution resulted in the growth of the copper and phosphorus amounts in the produced surface layer. The study on the effect of copper ions on the composition and structure of the surface layer created on titanium during the PEO process will be further continued. The aim is to explain and understand the mechanisms of the surface layer formation. Most likely, the top surface layer of PEO coatings consists mainly of Ti_3_(PO_4_)_4_∙nH_3_PO_4_ and Cu_3_(PO_4_)_2_∙nH_3_PO_4_ with a small addition of CuP_2_, CuO and Cu_2_O. Physical and mechanical properties, as well as those related to the biocompatibility, of these porous coatings will be the subject of our future works.

## Figures and Tables

**Table 1:** Descriptive statistics of the amount of copper in the surface layer formed on titanium after PEO.

Descriptive Statistics of Copper	10 g/L Cu(NO_3_)_2_		300 g/L Cu(NO_3_)_2_		600 g/L Cu(NO_3_)_2_	
	wt %	at %	wt %	at %	wt %	at %
Mean	1.7	1.2	2.4	1.6	12.2	7.6
Sta. Deviation	0.4	0.3	0.2	0.1	0.7	0.5
Median	1.6	1.2	2.4	1.6	12.1	7.5
Maximum	2.2	1.6	2.7	1.8	13.1	8.2
Minimum	1	0.7	2.2	1.4	10.6	6.6

**Table 2:** Descriptive statistics of the amount of phosphorus in the surface layer formed on titanium after PEO.

Descriptive Statistics of Phosphorus	10 g/L Cu(NO_3_)_2_		300 g/L Cu(NO_3_)_2_		600 g/L Cu(NO_3_)_2_	
	wt %	at %	wt %	at %	wt %	at %
Mean	8.9	13.2	67.3	40.3	42.9	54.9
Sta. Deviation	0.6	0.9	1.4	1.5	1.4	1.1
Median	9.1	13.5	67.8	39.9	42.8	55.1
Maximum	10.1	14.8	69.1	43.8	46.7	57.1
Minimum	7.8	11.6	64.2	38.6	41.2	53.3

**Table 3:** Descriptive statistics of the amount of titanium in the surface layer formed on titanium after PEO.

Descriptive Statistics of Titanium	10 g/L Cu(NO_3_)_2_		300 g/L Cu(NO_3_)_2_		600 g/L Cu(NO_3_)_2_	
	wt %	at %	wt %	at %	wt %	at %
Mean	89.4	85.6	67.3	58.1	45.2	37.5
Sta. Deviation	0.9	1.1	1.4	1.6	1.5	1.4
Median	89.4	85.7	67.8	58.6	45.1	37.4
Maximum	91.0	87.4	69.1	60.0	47.6	39.7
Minimum	87.9	83.7	64.2	54.6	43.3	35.4

**Table 4:** Chemical composition of PEO layers, at %.

Chemical Element	10 g Cu(NO_3_)_2_ in 1 L H_3_PO_4_	300 g Cu(NO_3_)_2_ in 1 L H_3_PO_4_	600 g Cu(NO_3_)_2_ in 1 L H_3_PO_4_
Titanium	7.0	5.0	4.9
Phosphorus	25.8 (16.8 at % in PO_4_^3−^)	24.9 (17.5 at % in PO_4_^3−^)	25.5 (17.3 at % in PO_4_^3−^)
Copper	0.2	0.3	0.4
Oxygen	67.0	69.8	69.2

**Table 5:** Fitting results of Cu 2p_3/2_ high resolution XPS spectra: BE—Binding Energy, FWHM—Full width at half maximum.

**300 g/L Cu(NO_3_)_2_**	BE, eV	931.3	932.9	934.1	935.1	936.1	937.2	938.1
	FWHM	0.6	1.3	1.1	1.1	1.0	1.2	0.5
	at %	3.6	26.6	25.0	18.7	15.3	8.9	1.9
**600 g/L Cu(NO_3_)_2_**	BE, eV	931.3	932.4	933.6	934.8	936.1	937.2	938.1
	FWHM	1.0	1.1	1.4	1.3	1.3	0.6	1.0
	at %	6.3	13.4	25.5	34.0	13.5	3.3	4.0

**Table 6:** Cu/P, Cu/Ti and P/Ti ratios gained on the basis of XPS results.

Ratio (by at %)	10 g Cu(NO_3_)_2_ in 1 L H_3_PO_4_	300 g Cu(NO_3_)_2_ in 1 L H_3_PO_4_	600 g Cu(NO_3_)_2_ in 1 L H_3_PO_4_
Cu/P	0.8	1.2	1.6
Cu/Ti	2.8	6.0	8.3
P/Ti	3.6	5.0	5.3
